# Methamphetamine Induces TET1- and TET3-Dependent DNA Hydroxymethylation of *Crh* and *Avp* Genes in the Rat Nucleus Accumbens

**DOI:** 10.1007/s12035-017-0750-9

**Published:** 2017-08-25

**Authors:** Subramaniam Jayanthi, Betina Gonzalez, Michael T. McCoy, Bruce Ladenheim, Veronica Bisagno, Jean Lud Cadet

**Affiliations:** 1 Molecular Neuropsychiatry Research Branch, NIH/NIDA Intramural Research Program, National Institutes of Health, 251 Bayview Boulevard, Baltimore, MD USA; 2 Instituto de Investigaciones Farmacológicas (Universidad de Buenos Aires–Consejo Nacional de Investigaciones Científicas y Técnicas), Ciudad Autónoma de Buenos Aires, Buenos Aires, Argentina; 3 Molecular Neuropsychiatry Research Branch, DHHS/NIH/NIDA IRP, 251 Bayview Boulevard, Baltimore, MD 21224 USA

**Keywords:** DNA methylation, DNA hydroxymethylation, TET, *Crh*, *Avp*, *Cartpt*, Neuropeptides

## Abstract

Methamphetamine (METH) addiction is a biopsychosocial disorder that is accompanied by multiple relapses even after prolonged abstinence, suggesting the possibilities of long-lasting maladaptive epigenetic changes in the brain. Here, we show that METH administration produced time-dependent increases in the expression of *corticotropin-releasing hormone* (*Crh*/*Crf*), *arginine vasopressin* (*Avp*), and *cocaine- and amphetamine-regulated transcript prepropeptide* (*Cartpt*) mRNAs in the rat nucleus accumbens (NAc). Chromatin immunoprecipitation (ChIP) assays revealed that METH increased the abundance of phosphorylated CREB (pCREB) at the promoter of *Cartpt* but not at *Avp* or *Crh* DNA sequences. In contrast, METH produced DNA hypomethylation at sites near the *Crh* transcription start site (TSS) and at intragenic *Avp* sequences. METH also increased DNA hydroxymethylation at the *Crh* TSS and at intragenic *Avp* sites. In addition, METH increased the protein expression of ten-eleven-translocation enzymes that catalyze DNA hydroxymethylation. Importantly, METH increased TET1 binding at the *Crh* promoter and increased TET3 binding at *Avp* intragenic regions. We further tested the role of TET enzymes in METH-induced changes in gene expression by using the TET inhibitor, 1,5-isoquinolinediol (IQD), and found that IQD blocked METH-induced increases in *Crh* and *Avp* mRNA expression. Together, these results indicate that METH produced changes in neuropeptide transcription by both activation of the cAMP/CREB pathway and stimulation of TET-dependent DNA hydroxymethylation. These results provide molecular evidence for epigenetic controls of METH-induced changes in the expression of neuropeptides.

**Electronic supplementary material:**

The online version of this article (10.1007/s12035-017-0750-9) contains supplementary material, which is available to authorized users.

## Introduction

Methamphetamine (METH) is an addictive psychostimulant whose abuse is widespread throughout the world. Similar to other psychostimulants, its rewarding properties are due to activation of the reward pathway that projects from the ventral tegmental area (VTA) to diverse brain structures including the nucleus accumbens (NAc) [1, 2]. Perturbations in this pathway may be involved in promoting relapses [3]. Importantly, because some neurotransmitters may be regulators of various addiction stages that include initiation, escalation, and recurrent relapses [4], pharmacological manipulations of these brain systems may offer opportunities to treat addiction [5, 6]. In fact, addiction appears to stimulate diverse pathways that may serve as a coincident signal to promote compulsive drug-taking behaviors [7, 8]. Some of these pathways may use neuropeptides that are known to respond to stress, as recently described in a microarray paper that documented METH-induced increases in the expression of *Crh*/*Crf*, *Avp*, and *Cartpt* in the NAc [8]. These peptides are known participants in stress responses in several brain regions [9, 10]. Indeed, the NAc is known to be activated by stress [11], possibly through the regulation of CRF since CRF-containing cell bodies are located in that structure [12, 13].

Exposure to psychostimulants has now been shown to result in epigenetic modifications in several regions of the brain [8, 14]. These epigenetic events include covalent histone modifications, DNA methylation, and expression of non-coding RNAs [15, 16]. Although most of these studies have investigated the effects of cocaine, a few reports have begun to identify a role for epigenetic mechanisms in METH-induced changes in the brain. For example, Ikegami et al. (2010) [17] showed that METH increased methylation of histone H3 at lysine 4 (H3K4me3) in the NAc. METH also produced differential effects on histone acetylation in the NAc, decreased histone deacetylase HDAC1 protein levels, and increased HDAC2 expression [18]. METH-induced conditioned place preference (CPP) increased histone H3 di- and trimethylation at lysine 4 (H3K4me2 and me3) but decreased H3K27me2 abundance [19]. Moreover, METH increased the expression of the DNA methyltransferase, DNMT1 [20, 21]. Interestingly, parental METH exposure produced DNA hypermethylation at 70 loci but DNA hypomethylation at 39 loci in the hippocampus of F1 mice [22]. More recently, we have shown that abstinence from METH SA is associated with increased hydroxymethylation in a subgroup of rats [23]. Taken together, these observations support the notion that METH may significantly alter biochemical pathways and behaviors via diverse epigenetic mechanisms including DNA methylation/demethylation processes.

DNA hypermethylation of cytosines adjacent to guanines (CpG sites) is thought to be responsible for transcription repression while DNA hypomethylation is more often associated with increased gene expression [24]. Demethylation is generated by the oxidation of 5-methylcytosine (5mC) to 5-hydroxymethylcytosine (5hmC) by ten-eleven translocation proteins [25] and by TET-dependent generation of 5-formylcytosine and carboxylcytosine followed by action of thymine-DNA glycosylase and base excision repair (BER) [26]. 5hmC is highly enriched in the adult brain [27] and appears to play a crucial role in the establishment of epigenetic states that promote gene expression and rapid behavioral adaptation [28].

As mentioned above, studies focusing on epigenetic neural adaptations in the NAc are important because that structure is an important link in the pathways that subsume drug-induced behavioral phenomena [29–31] including relapse [32]. Here, we report that METH increased *Crh* and *Avp* mRNA levels via increasing DNA hydroxymethylation around the *Crh* TSS and in *Avp* intragenic regions in the NAc. We found, in addition*,* that *Crh* TSS hydroxymethylation is mediated by TET1 while *Avp* hydroxymethylation is secondary to the actions of TET3. In contrast, METH-induced changes in *Cartpt* expression are mediated by increased binding of phosphorylated CREB (pCREB) at the *Cartpt* promoter. Together, these results support the notion that METH can produce a diversity of epigenetic changes in the NAc and identify DNA hydroxymethylation as a potentially important target for the development of anti-addictive drugs.

## Methods and Materials

### Animals and Drug Treatment

All animal treatments and procedures were approved by the National Institute of Drug Abuse Animal Care and Use Committee according to the *Guide for the Care and Use of Laboratory Animals* (ISBN 0-309-05377-3). Male Sprague-Dawley rats, 8–10 weeks old (Charles River Labs, Raleigh), weighing 250–300 g were housed in a humidity- and temperature-controlled (22.2 ± 0.2 °C) room with free access to food and water. The rats were group-housed (two rats per cage) with the regular light/dark schedule. All animals acclimated to the facility for 1 week. Following habituation, rats were randomly assigned to two groups (12 rats per group for each time point) and received intraperitoneal (i.p.) injections of saline or METH (10 mg/kg). This is a non-blinded study. To study the time-course effect of the METH injection, animals were euthanized at 1, 2, 3, or 4 weeks after the saline or drug injection. The euthanization procedure was completed at a consistent time of the day. NAc tissues were dissected and immediately frozen to be used in RT-qPCR, western blot analysis, methylcytosine DNA immunoprecipitation (MeDIP), and hydroxyMeDIP (hMeDIP) assays. For ChIP, MeDIP, and hMeDIP experiments, the NAc tissues were processed as described below.

For co-treatment with TET inhibitor, rats received an initial 1,5-isoquinolinediol (IQD) (3 mg/kg dissolved in DMSO, Santacruz) i.p. injection 4 h prior to either saline or to METH injections on day 1. Subsequently, IQD was injected on days 2, 4, and 6 days after the METH injection. During subsequent weeks (2nd, 3rd, and 4th week), two injections of IQD were given per week. The administered IQD dose was according to the published literature [33, 34]. This dose schedule resulted in four experimental groups (eight rats in each group): saline/saline (control); saline/METH; IQD (3 mg/kg)/saline (IQD + saline); IQD (3 mg/kg)/METH (IQD + METH).

### Quantitative PCR Analysis of mRNA Levels

Total RNA was isolated from one NAc hemisphere using RNeasy Mini kit (Qiagen) (*n* = 8–12 rats per group). Unpooled total RNA (0.5 μg) isolated from NAc samples was reverse-transcribed with oligo dT primers using Advantage RT-for-PCR kit (Clontech). RT-qPCR was performed essentially as described previously [18, 35] with Roche LightCycler 480 II (Roche Diagnostics) using iQ SYBR Green Supermix (BioRad). For all RT-qPCR experiments, individual data were normalized using the corresponding 18S mRNA level. The results are reported as fold changes calculated as the ratios of normalized gene expression data for METH-treated groups (at various time points) in comparison to the control group (SS). Primers for *Crh* (forward sequence 5′-CCT CGC AGA ACA GTG-3′ and reverse sequence 5′-CCT CAG AAG GTG GAA GGT G-3′), *Crhr1* (forward sequence-5′-GTC CCT GAC CAG CAA TGT TT-3′ and reverse sequence-5′-CGG AGT TTG GTC ATG AGG AT-3′), *Crhr2* (forward sequence-5′-AAG GTC CTA GGA GTG ATC CGA TT-3′ and reverse sequence-5′-GGA GCC ACC AGA GAG TGC AG-3′), *Avp* (forward sequence-5′-GAG TGT CGA GAG GGT TT-3′ and reverse sequence-5′-GGC GAT GGC TCA GTA GA-3′), *Avpr1a* (forward sequence-5′-CGT GGG TCC CTT TCA TA-3′ and reverse sequence-5′-GAA ATT CTC ATC CCA GAC T-3′), *Avpr1b* (forward sequence-5′-GGC CTA CAT CAC CTG GA-3′ and reverse sequence-5′-GCA GAA GAT GAG GAC TTA T-3′), and *Cartpt* (forward sequence-5′-TGG GAA GAA GAG GGA CT-3′ and reverse sequence-5′-TAA TTT GCA CAT GCT TCC A-3′) were synthesized at the Synthesis and Sequencing Facility of Johns Hopkins University (Baltimore, MD).

### Immunoblot Analysis

Western blot analyses were carried out from NAc protein lysates (*n* = 6 rats per group). Samples were homogenized separately in 10% *w*/*v* of ice-cold 10 mM HEPES buffer (pH 7.4) containing 10 mM KCl, 1.5 mM MgCl_2,_ 1%-Igepal CA-630 supplemented with a Roche protease inhibitor cocktail tablet (Roche Diagnostics). The homogenate was centrifuged for 5 min at 1400 × g to pellet nuclear fraction. The nuclear fraction was re-suspended in a nuclear lysis buffer (20 mM HEPES, 25% glycerol, 840 mM NaCl, 1.5 mM MgCl_2,_ 0.4 mM EDTA). The supernatant was the cytosolic fraction. Protein concentrations of the nuclear fractions were determined by a BCA assay (ThermoFisher Scientific) and were then denatured with sample buffer (62.5 mM Tris-HCl, 10% glycerol, 2% SDS, 0.1% bromophenol blue, and 50 mM DTT) at 100 °C for 5 min and subjected to SDS-PAGE. Proteins were electrophoretically transferred to Hybond-PVDF membrane (Amersham). Subsequently, the membranes were incubated overnight at 4 °C with specific antibodies against CREB and pCREB (Cell Signaling, #9197 and #9191), DNMT1 (Abcam, ab54759), DNMT3A, DNMT3B (Cell Signaling, #3598 and #2161), TET1, TET2, and TET3 (Santa Cruz, sc163443, sc136926, and sc139186). After incubation, the blots were washed with TBS containing 0.1% Tween-20. The membranes were then incubated with HRP-conjugated anti-rabbit, anti-mouse, or anti-goat secondary antibody for 1 h at room temperature. To confirm equal protein loading, the blots were reprobed with *α*-tubulin antibody (1:4000, 2 h at room temperature; Sigma). ECL plus chemiluminescent reagents (GE Healthcare) were used to detect protein expression. Signal intensity was measured with Carestream Molecular Imaging software. Experiments were done twice.

### Chromatin Immunoprecipitation Assay

NAc tissue was rapidly removed from rat brains (*n* = 8–10 rats per group), minced to ~ 1-mm-sized pieces, and immediately cross-linked in 1% formaldehyde for 15 min at room temperature. The cross-linking reaction was stopped by adding glycine to a final concentration of 0.125 M. The tissue was washed five times in cold PBS containing a Roche protease inhibitor cocktail tablet (Roche Diagnostics) and 1 mM PMSF (Sigma). Tissues were then rapidly frozen on dry ice. The fixed NAc tissues were suspended in SDS lysis buffer (Millipore) containing the Roche protease inhibitor cocktail (Roche Diagnostics) and 1 mM PMSF and homogenized twice for 10 s. Each sample was transferred to TPX plastic tube (Diagenode) and sonicated (15 cycles, 30″ ON and 30″ OFF) using the Diagenode Bioruptor device. Afterwards, fragmentation was checked by gel analysis to confirm a sheared range of 300–600 bp. Dynabeads (Life Technologies) were incubated with 5 μg of ChIP antibodies directed against pCREB (Cell Signaling, #9191), TET1 (ActiveMotif, #61443), TET2 (Millipore, MABE462), and TET3 (Santa Cruz, sc139186). As a control, NAc tissues were also incubated with 5 μg nonimmune rabbit IgG (Millipore, 12–370) overnight at 4 °C.

Equal amounts of chromatin lysate (60 μg) were diluted with ChIP dilution buffer (Millipore) to a final volume of 1.5 mL, and 100 μL of the pre-immunoprecipitated lysate was saved as “input” for later normalization. The chromatin lysate was then immunoprecipitated with appropriate antibodies overnight at 4 °C. The beads were sequentially washed once with low salt, high salt, LiCl, and Tris-EDTA according to the manufacturer’s instructions. The DNA-protein complexes were then eluted from the beads with 500 μL of NaHCO_3_/SDS elution buffer. DNA and proteins were dissociated at 65 °C for 4 h under high-salt conditions, followed by RNase A treatment for 30 min at 37 °C and proteinase K treatment for 1 h at 55 °C. The DNA was then extracted with phenol/chloroform, precipitated with ethanol, and finally resuspended in 80 μL of 10 mM Tris pH 8.0.

qPCR was performed with ChIP specific primers for *Crh*, *Avp*, and *Cartpt* (see Table S1 for details), using Roche LightCycler (Roche Diagnostics) and with iQ SYBR Green (BioRad) monitoring. All PCR reactions were performed in duplicate and included negative controls (no DNA). LightCycler software was used to calculate standard curves calculated using serial dilutions (100–0.1 ng) of input genomic DNA.

### Methylated DNA Immunoprecipitation and Hydroxymethylated DNA Immunoprecipitation

Genomic DNA was isolated from NAc tissue (*n* = 10 rats per group) by overnight proteinase k treatment, phenol-chloroform extraction, ethanol precipitation, and RNase digestion. Subsequently, 300 μL fractions of DNA (20 μg) were sheared by ultrasonic treatment using the Diagenode Bioruptor (12 cycles, 30 s “ON”, 30 s “OFF”) to obtain a fragment size between ~ 200–600 bp. After denaturation (10 min at 95 °C), 5 μg DNA was then immunoprecipitated overnight at 4 °C using 5 μL of mouse monoclonal anti-5mC antibody (Millipore, MABE146) for MeDIP assay or 5 μL of rabbit polyclonal anti-5hmC antibody (ActiveMotif, #39769) for hMeDIP assay in a final volume of 500 μL IP buffer (10 mM sodium phosphate (pH 7.0), 140 mM NaCl, 0.05% Triton X-100). We incubated the mixture with 80 μL of Dynabeads (Life Technologies) overnight at 4 °C and washed it three times with 700 μL of IP buffer. We then treated the beads with proteinase K for 3 h at 50 °C and recovered the methylated or hydroxymethylated DNA by phenol-chloroform extraction followed by ethanol precipitation. Sheared “input” DNA samples were collected prior to immunoprecipitation for subsequent comparison with immunoprecipitated DNA.

### qPCR on MeDIP and hMeDIP Samples

We carried out qPCR reactions with 20 ng of input DNA and immunoprecipitated methylated or hydroxymethylated DNA. For qPCR reactions, we used the iQ SYBR Green PCR master mix (BioRad) and Roche thermal cycler (Roche Diagnostics). The primer sequences and location are shown in Table S1. Reactions were done in duplicates and standard curves were calculated on serial dilutions (100–0.1 ng) of input genomic DNA. To evaluate the relative enrichment of target sequences after MeDIP or hMeDIP, we calculated the ratios of the signals in the immunoprecipitated DNA versus input DNA.

### Statistical Analysis

All the quantitative data are presented as mean + SEM. For time-course data comparing control and METH-treated groups at various time points, as well as for the experiment involving IQD (TET inhibitor co-treatment) and one-way ANOVA followed by Fisher’s PLSD, post hoc analysis was used to calculate significance for MeDIP, hMeDIP, and ChIP experiments, the data were assessed by unpaired Student’s *t* test (Stat View version 4.02). For all experiments, the null hypothesis was rejected at *p* < 0.05.

## Results

### METH Causes Long-Lasting Increases in Gene Expression of *Crh*, *Avp*, and *Cartpt* Neuropeptides

**Fig. 1:**
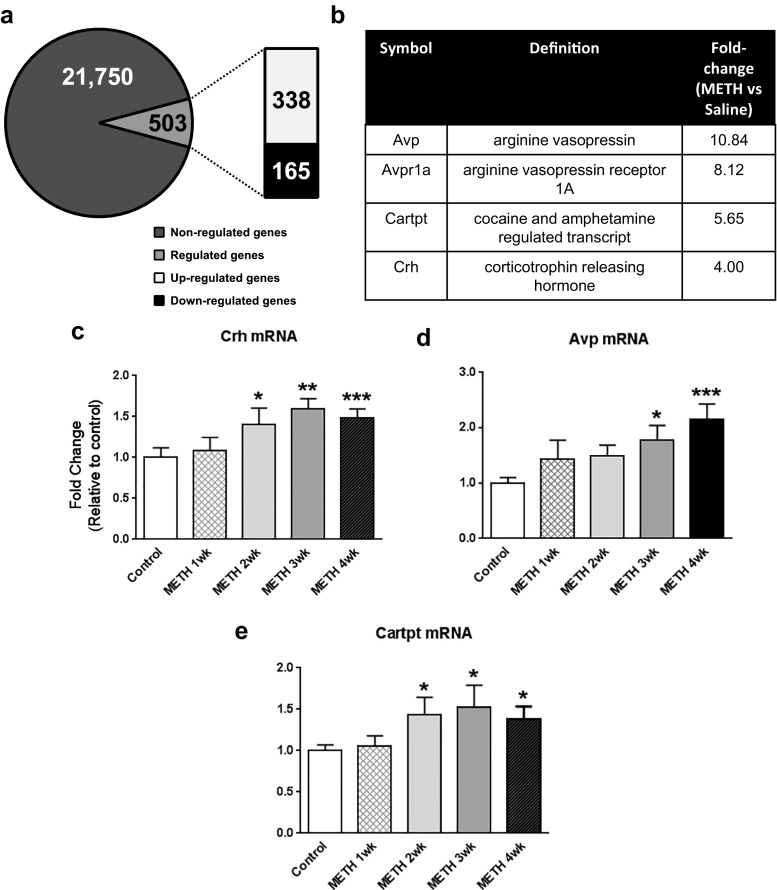
**a** Rats were treated with saline or METH (10 mg/kg), and microarray analyses (*n* = 6 rats per group) were performed 30 days later. The microarray data has been deposited in the NCBI database: GEO accession number GSE46717. **b** List of stress-related neuropeptide genes whose mRNA was significantly increased (greater than + 1.8-fold, *p* < 0.01) include *Avp* (10.8-fold), *Avpr1a* (8.12-fold), *Cartpt* (5.65-fold), and *Crh* (4-fold). METH produces time-dependent increased expression of **c** *Crh*, **d** *Avp*, and **e** *Cartpt*. Total RNA was extracted from the NAc (*n* = 8–12 rats per group), and quantitative PCR for *Crh*, *Avp*, and *Cartpt* was carried out as described in the text. The relative amounts of mRNA were normalized to 18S and quantified. Values represent means ± SEM of fold changes relative to the controls. Analysis of variance (ANOVA) followed by Fisher’s PLSD (protected least significance difference) was used to determine statistical significance. Key to statistics: *p < 0.05; **p < 0.01; and ***p < 0.001, in comparison to the control group

Injection of METH (10 mg/kg) produced substantial changes in gene expression in rats euthanized 1 month later, with 338 genes being upregulated and 165 being downregulated (Fig. 1a). Among these genes were stress-related *Crh*, *Avp*, and *Cartpt* that showed increased expression [36] (Fig. 1b). To measure potential METH-induced time-dependent changes in *Crh*, *Avp*, and *Cartpt* mRNA levels, we injected the rats with either saline or METH and euthanized the animals at 1, 2, 3, or 4 weeks after the saline or METH injection. Figure 1 shows the time course of *Crh* (Fig. 1c), *Avp* (Fig. 1d), and *Cartpt* (Fig. 1e) expression after the drug injection. METH caused time-dependent increases in the mRNA levels of all three genes. The increases in the expression of *Crh* [F(4,47) = 4.837, *p* = 0.0024] and *Cartpt* injection [F(4,51) = 2.727, *p* = 0.0392] were apparent after 2 weeks and remained elevated for the duration of the study (Fig. 1c and e). METH-induced increases in *Avp* expression were significant at 3 and 4 weeks after the injection [F(4,44) = 4.577, *p* = 0.0035] (Fig. 1d). We also measured mRNA levels of *Crh* and *Avp* receptors. Supplemental Fig. S1 shows these results. *Crhr1* [F(4,48) = 4.134, *p* = 0.0059], *Crhr2* [F(4,42) = 4.892, *p* = 0.0025], and *Avpr1a* [F(4,45) = 4.213, *p* = 0.0056] mRNA levels showed significant increases whereas *Avpr1b* [F(4,53) = 2.571, *p* = 0.0483] showed significant decrease at 1 and 4 weeks after METH injection (Fig. S1).

### METH Increases Enrichment of pCREB on *Cartpt* Gene Promoter

**Fig. 2:**
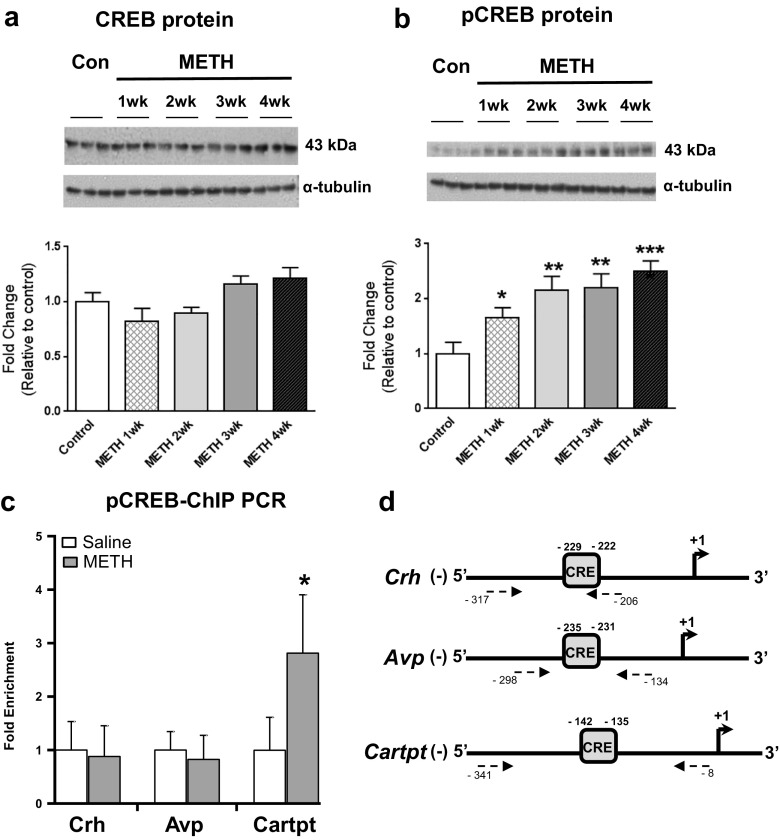
METH increased CREB phosphorylation and pCREB abundance at the *Cartpt* gene promoter. Western blot analyses (*n* = 6 rats per group) revealed no METH-induced changes in **a** CREB protein levels but significant time-dependent increases in the abundance of **b** pCREB. Representative photomicrographs show results of three samples per time point. For quantification, the signal intensity was normalized to *α*-tubulin. Values represent means ± SEM of fold changes relative to the controls. Statistical analysis was performed as in Fig. 1. Key to statistics: *p < 0.05; **p < 0.01; and ***p < 0.001 in comparison to the control group. For ChIP assays, we used animals euthanized at the 30-day time points. ChIP assays were performed using an anti-pCREB (cell signaling), and quantitative PCR was performed as described in the text using specific ChIP primers directed at *Crh*, *Avp*, or *Cartpt* **c** promoter regions. **d** Primers used for PCR following ChIP using antibodies against pCREB are pictured in dotted arrows and were designed so that resulting PCR amplicons contain the corresponding CRE site. CRE site sequence: TGACGTCA. In *Avp* promoter, the CRE site presents only half of the binding sequence. CRE half-site sequence: CGTCA (see Table S1 for primer sequences). Values represent means ± SEM of fold changes relative to the controls. Unpaired Student’s *t* test was used for statistical analyses. Key to statistics: *p < 0.05; **p < 0.01; and ***p < 0.001 in comparison to the control group

Psychostimulant-induced changes in gene expression are mediated, in part, by a dopamine-cAMP-CREB (Cyclic AMP Response Element-Binding) pathway that involves CREB phosphorylation [37, 38]. CREB is also involved in regulating stress-induced gene expression [7]. We thus wondered if the injection of METH might increase phosphorylated CREB (pCREB) in a time-dependent fashion. To test this idea, we used specific antibodies to measure CREB and pCREB protein abundance and found no changes in CREB protein levels (Fig. 2a) but time-dependent increases in pCREB abundance in NAc nuclear fractions (Fig. 2b). The initial increase in pCREB abundance was apparent by 1 week (~ 1.7-fold) and reached 2.5-fold after 4 weeks injection [F(4,20) = 7.019, *p* = 0.0011]. We next tested if METH might increase pCREB recruitment onto *Crh*, *Avp*, and *Cartpt* CRE DNA sequences by performing ChIP-qPCR assays. Figure 2c illustrates that METH increased pCREB binding on the *Cartpt* gene promoter region but not on *Crh* or *Avp* CRE DNA sequences (see Table S1 for primer details).

### DNA Methylation and Hydroxymethylation Regulate METH-Induced Expression of *Crh* and *Avp*

**Fig. 3:**
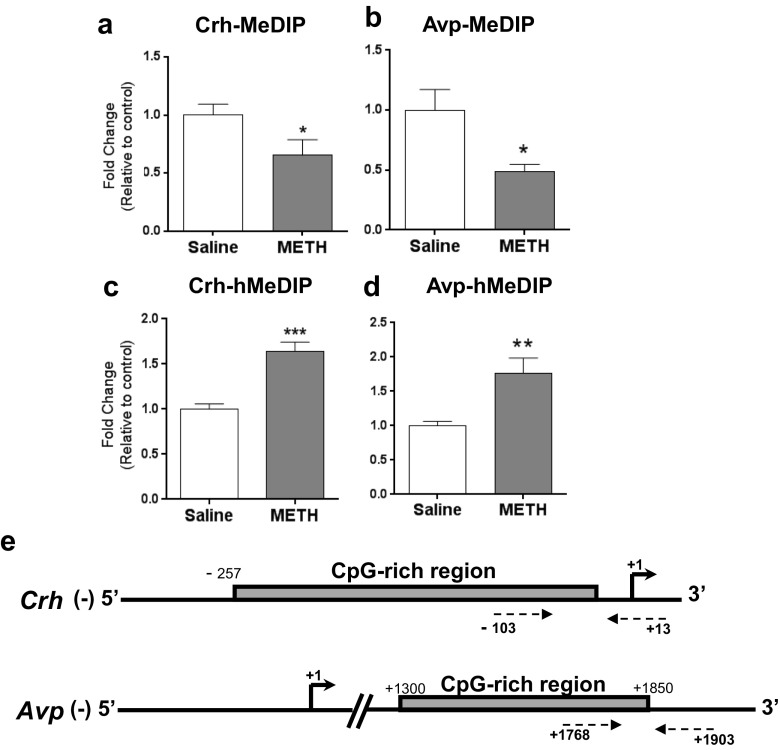
METH caused DNA hypomethylation (**a** and **b**) and increased DNA hydroxymethylation (**c** and **d**) at CpG-rich sites near the promoter of *Crh* and at CpG-rich *Avp* intragenic sites. For MeDIP and hMeDIP assay, we used animals euthanized at the 30-day time point (*n* = 10 rats per group). Antibodies directed against either 5mC (Millipore) or 5hmC (ActivMotif) were used to precipitate methylated or hydroxymethylated DNA. Relative enrichment of 5mC or 5hmC was calculated using real-time PCR with specific primers directed at the promoter region of *Crh* and at the intragenic *Avp* CpG rich site (+ 1.8Kb). **e** Primers used for MeDIP and hMeDIP assay are pictured in dotted arrows (see Table S1 for primer sequences). Values represent means ± SEM of fold enrichment relative to controls. Statistical significance was determined by unpaired Student’s *t* test. Key to statistics: *p < 0.05; **p < 0.01 in comparison to the control group

**Fig. 4:**
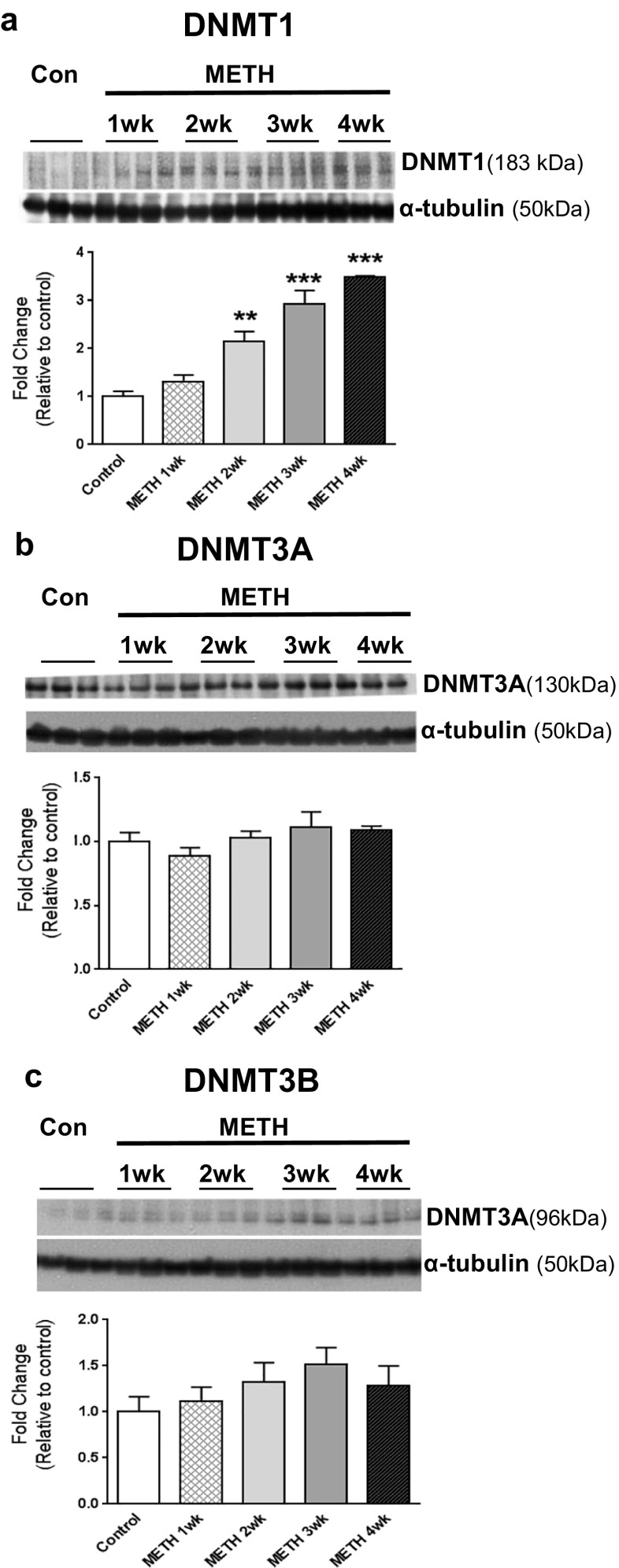
METH causes time-dependent increased DNMT1 protein expression. Western blot analyses (*n* = 6 rats per group) showed significant time-dependent increases in the protein levels of **a** DNMT1 and no change in **b** DNMT3A and **c** DNMT3B. Representative photomicrographs show results of three samples per time point. For quantification, the signal intensity was normalized to *α*-tubulin. Values represent means ± SEM of fold changes relative to the controls. Statistical significance for the different time points was compared by analysis of variance followed by Fisher’s PLSD. Key to statistics: *p < 0.05; **p < 0.01; ***p < 0.001 vs. control group

Because pCREB did not appear to play a role in *Crh* and *Avp* mRNA expression, we wondered if METH-induced DNA hypomethylation might be involved in the upregulation of these mRNAs. Analysis of METH-induced effects on DNA methylation status of the *Crh* and *Avp* genes by using MeDIP-qPCR assays revealed significant METH-induced DNA hypomethylation at the *Crh* promoter region [*t* = 2.209, *p* = 0.049] (Fig. 3a) and at a CpG-rich region within the *Avp* gene body [*t* = 2.615, *p* = 0.024] (Fig. 3b). However, when we measured the protein expression of DNA methyltransferases (DNMT1, DNMT3A, and DNMT3B) [39], we found that METH increased the protein expression of DNMT1 [F(4,20) = 35.897, *p* < 0.0001] but not the expression of DNMT3A and DNMT3B (Fig. 4a–c). Although these results are consistent with previous findings showing METH-induced increases in DNMT1 [20, 21], they suggested that the METH-induced DNA hypomethylation was most likely not secondary to decreased DNMT expression but probably to TET-induced DNA hydroxymethylation. TET proteins convert 5mC to 5hmC and 5fC (5-formyl cytosine) and 5caC (5-carboxyl cytosine) [25]. DNA hydroxymethylation is also known to be a stable epigenetic mark that regulates gene expression [40, 41]. We thus tested the possibility that METH might cause increased DNA hydroxymethylation in the Nac. We indeed detected drug-induced increased hMeDIP enrichment at the *Crh* TSS [*t* = − 5.618, *p* < 0.0001] and the *Avp* intragenic region [*t* = − 3.509, *p* = 0.0038] (Fig. 3c and d).

### TET Proteins Mediate METH-Induced Alterations in DNA Hydroxymethylation

**Fig. 5:**
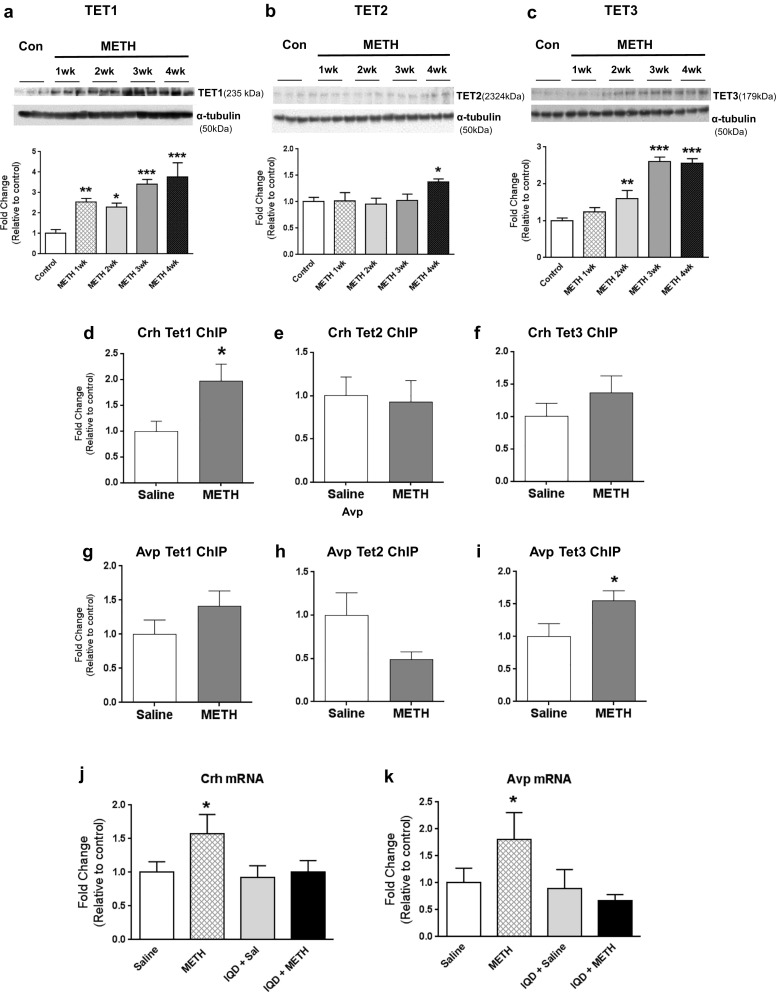
TET1 and TET3 respectively regulate METH-induced DNA hydroxymethylation at *Crh* and *Avp* DNA sequences (*n* = 8–10 rats per group). Western blot analyses (*n* = 6 rats per group) revealed significant METH-induced time-dependent changes in the protein levels of TET1 (**a**) and TET3 (**c**). TET2 protein (**b**) levels were only increased at 4 weeks after the METH injection. Representative photomicrographs show results of three samples per group. For quantification, the signal intensity was normalized to *α*-tubulin. Values represent means ± SEM of fold changes relative to controls. ANOVA followed by Fisher’s PLSD was used to detect significance level. Key to statistics: *p < 0.05; **p < 0.01; ***p < 0.001, in comparison to the control group. METH increased enrichment of TET1 on the *Crh* gene promoter (**d**) but not on the *Avp* intragenic site (**g**). There was, however, increased TET3 binding at the *Avp* intragenic region but (**i**) not on the *Crh* promoter (**f**). TET2 binding was not affected by METH (**e**, **h**). Values represent means ± SEM of fold enrichment relative to the controls. Statistical significance was determined by unpaired Student’s *t* test. Key to statistics: *p < 0.05, in comparison to the control group. Co-treatment with TET protein inhibitor, 1,5-isoquinolinediol (IQD) (*n* = 8 rats per group), blocked the METH-induced increases in *Crh* (**j**) and *Avp* mRNA levels (**k**). Values represent means ± SEM of fold changes relative to the controls. Measurements of mRNA levels and statistical analysis are described in Fig. 1. Key to statistics: *p < 0.05 vs. control group

To test if TET proteins were involved in DNA hydroxymethylation, we measured TET1, TET2, and TET3 protein levels in nuclear fractions obtained from the NAc. There were significant time-dependent increases in TET1 and TET3 protein levels in the NAc after METH, with TET1 reaching 3.8-fold [F(4,20) = 9.055, *p* = 0.0005] (Fig. 5a) and TET3 reaching 2.5-fold increases [F(4,20) = 27.245, *p* < 0.0001] (Fig. 5c). TET2 expression only reached 1.4-fold [F(4,20) = 2.429, *p* = 0.0815] increases (Fig. 5b).

To further investigate the role of TET proteins in METH-induced DNA hydroxymethylation, we used ChIP-PCR with specific antibodies against the TET proteins to measure TET binding to *Avp* and *Crh* (Fig. 5d–i). We found significant METH-induced increased enrichment of TET1 on the *Crh* promoter sequence [*t* = − 2.500, *p* = 0.0245] (Fig. 5d) and increased enrichment of TET3 on *Avp* intragenic CpG-rich site [*t* = − 2.228, *p* = 0.0428] (Fig. 5i). There were neither significant changes in TET2 (Fig. 5e) and TET3 (Fig. 5f) binding at the *Crh* promoter nor in TET1 (Fig. 5g) and TET2 (Fig. 5h) binding at the *Avp* intragenic site.

So far, our observations had indicated that METH increased *Crh* and *Avp* expression by TET-dependent DNA hydroxymethylation. To further test the role of TET in this process, we used the TET inhibitor, IQD [34], that prevents DNA demethylation and the accumulation of TET-induced DNA intermediates [33]. IQD alone did not influence *Crh* and *Avp* expression. However, IQD co-treatment with the psychostimulant significantly blocked METH-induced increased *Crh* (Fig. 5j) and *Avp* (Fig. 5k) mRNA levels.

## Discussion

The present study shows that an injection of METH produced long-lasting alterations in the expression of *Crh*, *Avp*, and *Cartpt* mRNAs. These neuropeptides are involved in a diversity of neuroendocrine responses to stressful events [5, 42]. These results are consistent with the observations that a single injection of amphetamine (AMPH) can cause hypersecretion of plasma adrenocorticotropin hormone (ACTH) and corticosterone measured at 1 and 3 weeks [43]. We show, in addition, that METH increased pCREB binding at the *Cartpt* promoter but not at *Crh* and *Avp* promoters. In contrast, METH caused DNA hydroxymethylation at a CpG-rich region in the promoter of the *Crh* gene and at a CpG-rich intragenic site of the *Avp* gene. DNA hydroxymethylation is known to be catalyzed by TET enzymes [25]. Consistent with this fact, we also found that METH increased TET1 binding at the *Crh* promoter and TET3 binding at the *Avp* intragenic site. Importantly, TET inhibition with the drug, IQD, blocked METH-induced *Crh* and *Avp* mRNA overexpression. Our findings are consistent with the demonstration that METH-induced gene transcription is regulated by diverse epigenetic mechanisms that include post-translational histone modifications and DNA methylation [18, 20, 22, 38, 44].

The present observations further implicate a role of stress-related neuropeptides in the biochemical and molecular effects of drugs of abuse, at least, in the NAc. For example, the expression of *Crh*, *Avp*, and *Cart* is increased in various brain regions in response to a diversity of stressful events including administration of psychostimulants [42, 45–47]. Our observations of increased NAc *Crh* expression after the METH injection are consistent with previous studies that have reported increased *Crh* in several brain regions after withdrawal of several drugs of abuse [10, 48–50] including increased levels in the amygdala after METH withdrawal [51]. A potential role of *Crh* in METH-induced molecular and behavioral changes is supported by the fact that administration of CP-154-526, a *Crh* receptor 1 antagonist, interferes with METH-induced reinstatement of extinguished METH-seeking behavior [52]. In addition to *Crh*, we also found increases in *Avp* and *Cartpt* mRNA levels. *Avp* has previously been implicated in the acquisition of cocaine-seeking behavior [53] and cocaine withdrawal [54]. *Cart* peptide is also increased in reward-related brain subregions following administration of various psychostimulants [55–57]. Our results are also consistent with the report of increased *Cartpt* mRNA in the NAc of victims of cocaine overdose [58]. Increased *Cartpt* is thought to participate in the regulation of the biochemical effects of dopamine in the NAc [55–57]. This suggestion is supported by behavioral studies showing that injection of *Cart* peptide into the NAc reduces the rewarding effects of cocaine [59] and blunts AMPH-induced locomotor activity and sensitization [60, 61]. Although the behavioral effects of these neuropeptides have been well documented, the epigenetic mechanisms that control their expression remained to be clarified.

In this context, we first explore the potential role of CREB phosphorylation in the METH-induced increases in the expression of these genes because pCREB is known to regulate psychostimulant-induced gene expression [38, 62]. Other studies have also implicated pCREB in the regulation of *Crh*, *Avp*, and *Cartpt* expression [63–65]. Surprisingly, we found that pCREB binding was increased only on the *Cartpt* promoter, indicating that METH had increased *Cartpt* expression via the dopamine-PKA-pCREB pathway in a manner consistent with a direct dopaminergic input to *Cart*-peptide-containing neurons in the NAc [66]. In addition to CREB phosphorylation, recent studies have implicated DNA methylation in the regulation of *Crh* [67, 68] and of *Avp* [69] expression. The latter reports are consistent with our demonstration of METH-induced DNA hypomethylation on the *Crh* promoter and at the CpG-rich region within the *Avp* gene body. The drug-induced DNA hypomethylation is consistent with our observations of METH-induced increased DNA hydroxymethylation at the *Crh* and *Avp* DNA sequences (see Fig. 3). Importantly, DNA hydroxymethylation is thought to drive transcription of many genes [70, 71]. Hydroxymethylated DNA is very abundant in the brain where it coordinates transcriptional activity [27, 72]. Hydroxymethylcytosine (5hmC) is found in gene bodies and at intron-exon boundaries of synaptic plasticity-related genes [71, 73, 74]. Together, these facts suggest that this DNA marker may play an important role in the long-term effects of psychostimulant exposure including cocaine [75].

Our findings that METH increased TET1 binding on the *Crh* promoter and TET3 binding on the *Avp* intragenic region are of interest because they illustrate the possibility of differential binding of these enzymes at different loci of two genes that are co-regulated by METH. These observations are consistent with recent reports indicating that accumulation of 5hmC at promoter regions is driven by the actions of TET1 [76, 77] whereas intragenic of 5hmC is associated with the presence of TET3 [28, 78]. For example, overexpression of TET3 in mature olfactory neurons increased 5hmC levels in gene bodies that positively correlated with increased transcription [78]. Our pharmacological interventions with a TET inhibitor (IQD) that blocked the METH-induced changes in the expression of both *Crh* and *Avp*, suggesting this agent may be worth pursuing in animal models of addiction that includes stress-induced relapse to drug seeking. This statement is consistent with the report that IQD treatment can prevent impaired recognition memory measured in mice that have been exposed to a combination of stress and alcohol [79].

**Fig. 6:**
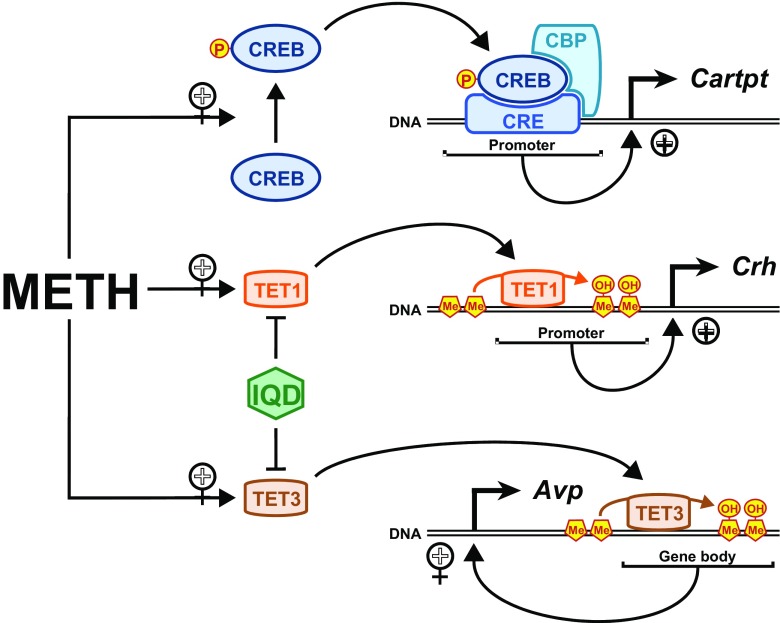
METH promotes diverse epigenetic modifications in the rat nucleus accumbens. The METH injection produced CREB phosphorylation and binding of pCREB on the CRE site on the *Cartpt* promoter and recruitment of the histone acetyl-transferase, CREB binding protein (CBP). This process leads to increased histone acetylation followed by increased *Cartpt* mRNA expression. On the other hand, METH increased TET1 binding at the *Crh* promoter and TET3 binding at an *Avp* intragenic site. The TET binding to these sequences promote DNA hydroxymethylation followed by increased *Avp* and *Crh* transcription. This statement is supported by the fact that the TET inhibitor, IQD, could prevent METH-induced increased *Crh* and *Avp* mRNA levels

In summary, we have shown, for the first time, that TET-dependent DNA hydroxymethylation is the main determinant of *Crh* and *Avp* regulation in the rat NAc after injection of METH. Our results also specify TET1 and TET3 as the respective enzymes involved in the catalytic steps that produce DNA hydroxymethylation at *Crh* and *Avp* DNA sequences. These results shed light on the epigenetic mechanisms that link METH to neuropeptide expression in the rat NAc. The scheme in Fig. 6 illustrates these mechanisms. Furthermore, our demonstration that a TET inhibitor can block the transcriptional effects of METH indicates that such an approach may help to expand our armamentarium against METH addiction. This suggestion will need to be tested by assessing if these effects of METH can be expanded to self-administration models of addiction. The role of other stress-responsive systems including the extended amygdala will also need to be investigated in future experiments.

## Electronic Supplementary Material

Fig. S1 (PPTX 81 kb)

Table S1 (DOCX 14 kb)
