# Supplementary figures and images for: Methamphetamine Induces TET1- and TET3-Dependent DNA Hydroxymethylation of Crh and Avp Genes in the Rat Nucleus Accumbens
Source: Mol Neurobiol. 2017 Aug 25;55(6):5154–66. doi: 10.1007/s12035-017-0750-9 (PMC5948251; doi:10.1007/s12035-017-0750-9)

## Slide 1
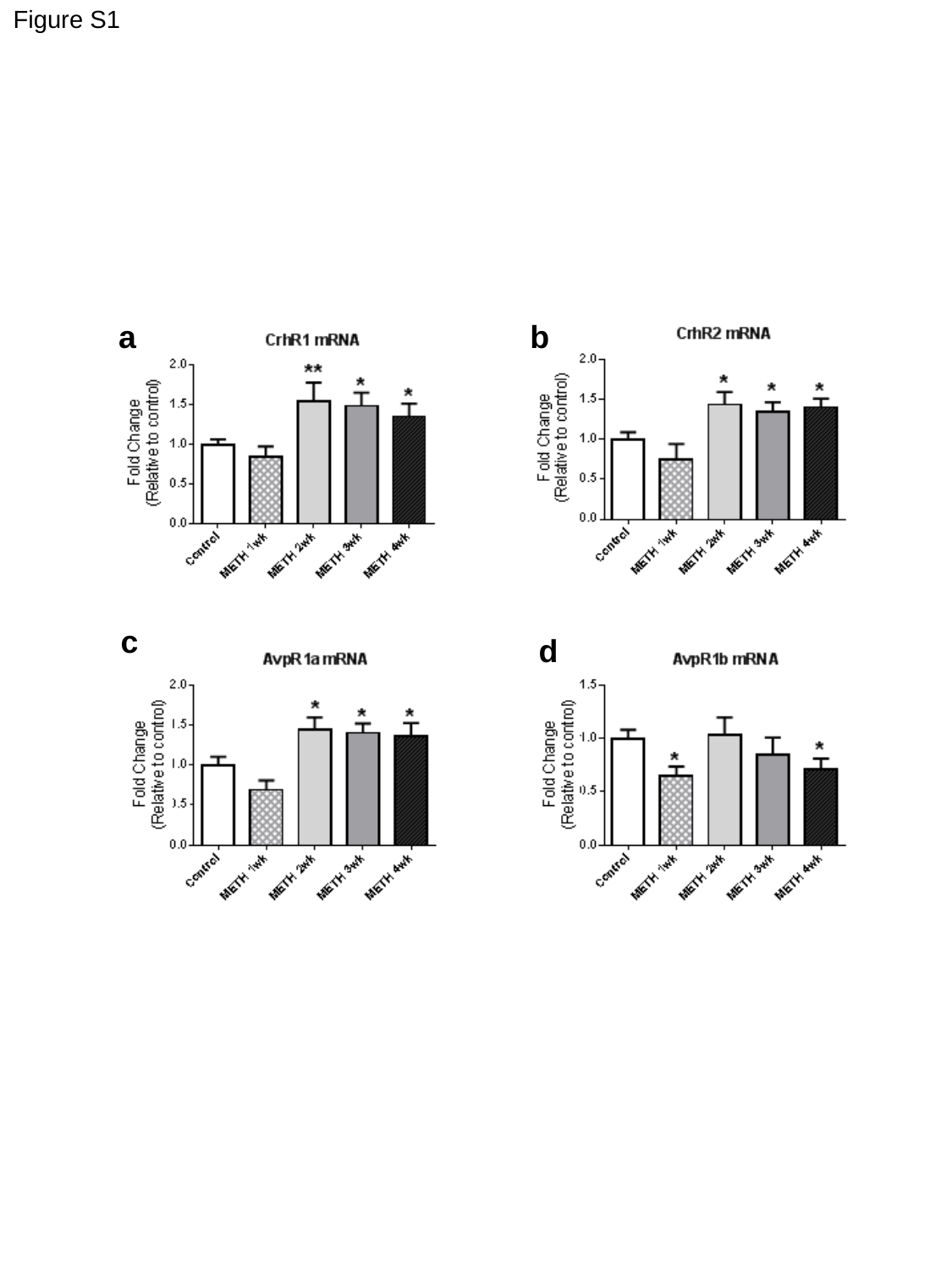

Figure S1
a
b
c
d

Supplement: Supplementary file 1 — (PPTX 81 kb) [file 12035_2017_750_MOESM1_ESM.pptx]
