# Supplementary material for: Methamphetamine Induces TET1- and TET3-Dependent DNA Hydroxymethylation of Crh and Avp Genes in the Rat Nucleus Accumbens
Source: Mol Neurobiol. 2017 Aug 25;55(6):5154–66. doi: 10.1007/s12035-017-0750-9 (PMC5948251; doi:10.1007/s12035-017-0750-9)
Supplement: Supplementary file 2 — (DOCX 14 kb) [file 12035_2017_750_MOESM2_ESM.docx]

**Table S1. PCR primers sequences and locations.**

| **Gene** | **Experiment** | **Forward primer (5’ to 3’)** | **Reverse primer (5’ to 3’)** |
| --- | --- | --- | --- |
| ***Cartpt*** | pCREB-ChIP | CCGAAGGCATTTTCCATTTC | CACTGCGCTCTCCCTCTTCT |
| ***Crh*** | pCREB-ChIP | TCAGTATGTTTTCCACACTTGGAT | TTTATCGCCTCCTTGGTGA |
|  | MeDIP | CACGCAATCGAGCTGTCAA | GCTCTGAGTTTCTCCACAC |
|  | hMeDIP |  |  |
|  | TET-ChIP |  |  |
| ***Avp*** | pCREB-ChIP | GGTGACCCTCAAGTCGG | AGCAGTGATTCAGGCATCT |
|  | MeDIP | CGAGTGTCGAGAGGGTTT | CAGAATCCACGGACTCTT |
|  | hMeDIP |  |  |
|  | TET-ChIP |  |  |
